# Walking Bout Detection for People Living in Long Residential Care: A Computationally Efficient Algorithm for a 3-Axis Accelerometer on the Lower Back

**DOI:** 10.3390/s23218973

**Published:** 2023-11-04

**Authors:** Mhairi K. MacLean, Rana Zia Ur Rehman, Ngaire Kerse, Lynne Taylor, Lynn Rochester, Silvia Del Din

**Affiliations:** 1 Department of Biomechanical Engineering, Faculty of Engineering Technology, University of Twente, 7522 LW Enschede, The Netherlands; 2 Translational and Clinical Research Institute, Faculty of Medical Sciences, Newcastle University, Newcastle upon Tyne NE2 4HH, UK; rrehman5@its.jnj.com (R.Z.U.R.); lynn.rochester@newcastle.ac.uk (L.R.); 3 School of Population Health, Faculty of Medical and Health Sciences, University of Auckland, Auckland 1023, New Zealand; n.kerse@auckland.ac.nz (N.K.); lm.taylor@auckland.ac.nz (L.T.); 4 The Newcastle upon Tyne Hospitals NHS Foundation Trust, Newcastle upon Tyne NE7 7DN, UK; 5 National Institute for Health and Care Research (NIHR), Newcastle Biomedical Research Centre (BRC), Newcastle University and The Newcastle upon Tyne Hospitals NHS Foundation Trust, Newcastle upon Tyne NE2 4HH, UK

**Keywords:** wearable sensors, locomotion, algorithm design, accelerometer, older adults

## Abstract

Accurate and reliable measurement of real-world walking activity is clinically relevant, particularly for people with mobility difficulties. Insights on walking can help understand mobility function, disease progression, and fall risks. People living in long-term residential care environments have heterogeneous and often pathological walking patterns, making it difficult for conventional algorithms paired with wearable sensors to detect their walking activity. We designed two walking bout detection algorithms for people living in long-term residential care. Both algorithms used thresholds on the magnitude of acceleration from a 3-axis accelerometer on the lower back to classify data as “walking” or “non-walking”. One algorithm had generic thresholds, whereas the other used personalized thresholds. To validate and evaluate the algorithms, we compared the classifications of walking/non-walking from our algorithms to the real-time research assistant annotated labels and the classification output from an algorithm validated on a healthy population. Both the generic and personalized algorithms had acceptable accuracy (0.83 and 0.82, respectively). The personalized algorithm showed the highest specificity (0.84) of all tested algorithms, meaning it was the best suited to determine input data for gait characteristic extraction. The developed algorithms were almost 60% quicker than the previously developed algorithms, suggesting they are adaptable for real-time processing.

## 1. Introduction

Measurement of walking activity in long-term residential care environments is important for understanding mobility function, assessing the effects of interventions, tracking disease progression, monitoring the risk of falling, and identifying unmet mobility needs [1,2,3]. People living in long-term-care homes often have mobility issues, which are typically the result of aging or neurological or musculoskeletal disorders. The risk of falling for people living in long-term care is substantially higher than for people living in the community, with the average fall rate for people living in long-term care found to be between 1.5 and 2.75 times per year [4,5,6]. Falls are a common cause of many problems including bone fractures, loss of confidence in mobility, and high healthcare costs [7,8]. Many countries predict a growing population of older adults, which in turn predicts an increased incidence of mobility-impacting diseases and conditions [9,10,11,12]. Although we can measure and assess gait in controlled lab environments, there are numerous benefits to measuring walking in habitual environments or the “real-world” [13,14,15,16]. Measures of walking in the real world give a more truthful representation of a person’s walking ability, which usually differs from the walking ability displayed in the lab. Furthermore, real-world walking measurements can be used to evaluate the activity level, physical independence, and fall risk of a person.

To quantify gait characteristics for medically or socially relevant insights it is imperative to first accurately identify when a person is walking. Algorithms can process walking data to determine macro and micro gait metrics [17]. Macro gait metrics include walking volume, pattern, and variability, whereas micro-level outcomes include pace, rhythm, variability, asymmetry, and postural control. The gait metrics can be used to diagnose disease (and disease progression), evaluate risk, and determine suitable treatments. It is therefore important that the gait characteristics are accurate, which in turn, means that the walking bout data used to extract gait characteristics must be accurate. Attempting to extract gait characteristics from non-walking data will produce nonsensical results which, when included with gait characteristics derived from actual walking, confounds the overall results and findings [15,18]. As such, high specificity in identifying walking bouts is vital to accurate and reliable outcomes.

Many gait identification algorithms have been developed for wearable sensors such as accelerometers and inertial measurement units (IMU) to isolate walking bouts in the real-world [15,19,20]. IMUs consist of a 3-axis linear accelerometer and 3-axis gyroscope. The gyroscope data provides more information on body motion and orientation, which is useful for identifying gait. However, gyroscopes have a substantially higher power consumption than accelerometers, which reduces the battery life of the wearable sensor [21]. Furthermore, incorporation of gyroscope data increases the complexity of the algorithm due to sensor fusion. Some walking activity algorithms use IMUs on each of the lower body segments and, in addition to identifying walking, can produce a full kinematic analysis of gait. Algorithms have also been developed for use with a limited sensor set, such as: one IMU on the thigh [22,23]; one IMU on a foot [24,25]; and one 3-axis accelerometer on the lower back [26,27]. Limited sensor sets have quicker set-up times and reduce the burden on the user, which is particularly important for older persons and those who have age-related diseases. A recent study found that wearable sensors on the lower back had high comfort and acceptability for a varied clinical population [28]. It is important to note that the exact position and orientation of the sensor(s) impacts the recorded signal and therefore the quality of the results [29]. Analytical pipelines of concurrent and sequential algorithms designed for these set-ups can identify multiple gait characteristics like walking speed, gait asymmetry, and stride length. Most of these algorithms have been developed and validated for a specific target population such as healthy young adults, older people, people with Parkinson’s disease, or cerebral palsy [30,31,32,33]. Due to differences in gait characteristics, mobility capacity and performance, algorithms designed for a specific population are not necessarily suitable for a different population.

Identifying walking bouts for people living in long-term residential care is challenging due to generally low activity, slow walking speeds, halting gait patterns, short walking bouts, and reliance on walking aids which can alter gait characteristics and acceleration patterns [34,35]. The population of people living in long term care is heterogeneous, with many different conditions manifesting a variety of mobility impairments [36,37]. In general, most residents’ gait can be characterized by slow walking. Slower walking speeds are challenging for previously developed algorithms, with slower speeds generally producing more errors in walking bout detection [15]. Furthermore, cognitive impairment, which can impact mobility, is quite present in this population [38]. No walking detection algorithm has yet been developed or validated on this population.

A variety of approaches are employed in gait detection algorithms, including generic, personalized, and machine learning techniques. Generic algorithms are “out-of-the-box” or standard designs that can be used immediately within the target population. At most, the height or leg length of the person is needed to derive spatial gait characteristics (i.e., step or stride length). Personalized algorithms incorporate some characteristic(s) of the individual in the processing which requires a form of calibration [39,40,41]. The most complex variety of walking detection algorithms are those that use machine learning to improve their walking bout detection accuracy [42,43,44,45]. Although the machine learning algorithms can be accurate, they also require a substantial amount of time and training data to be useful. Furthermore, they have comparatively higher computational power requirements when compared to non-machine learning algorithms. For off-line data processing, high computational expense can be managed by powerful computers or cloud-based computing. However, low computational costs are very beneficial for on-line processing, allowing the sensor and processor to be packaged in one wearable device and producing real-time, useable outputs such as predicting and alerting the wearer of an imminent risk of falling.

Walking bout detection algorithms based on accelerometer data from the lower back have been developed and tested in a variety of populations with promising results. In 2014, Iluz et al. developed a walking bout detection algorithm for people with Parkinson’s disease [18]. They band pass filtered the acceleration signals between 0.5 and 3 Hz with the assumption that gait typically occurs between these frequencies. A 5-s running window on the vertical and anterior–posterior acceleration was convoluted with a 2 Hz sinusoidal signal, for which the local maxima in the resultant signal represented a gait cycle. Windows containing 2–15 steps were classified as gait. The accuracy of this algorithm at detecting walking bouts was not reported, but was later evaluated against gold standard lab based data collection through the Mobilise-D consortium which found a specificity above 0.94 in multiple cohorts including Parkinson’s disease, multiple sclerosis, and healthy older adults [15]. Paraschiv-Ionescu et al. later developed a walking detection algorithm based on the magnitude (or 3D norm) of the acceleration signal which was low pass filtered [46]. The improved version of their algorithm, developed a year later, employed multiple smoothing and enhancement stages to the acceleration magnitude [47]. Peaks in the signal above a fixed threshold in the original algorithm, or a personalized threshold in the revised version, were considered heel strikes. The time between heel strikes was compared to a second adaptive threshold to determine steps and thus walking bouts. For a cohort of stroke patients, the specificity of the original and revised algorithms were 0.85 and 0.93, respectively. Both the Paraschiv-Ionescu et al. algorithms had specificity above 0.94 in the cohorts examined through Mobilise-D [15]. Although the state-of-the-art walking bout detection algorithms discussed here have high sensitivity, we identify two limitations. The first is that these algorithms have not been validated in a very slow-walking population, such as people living in long-term care homes. Secondly, there is no information on the computational expense of these algorithms. We can assume that windowing and repeated filters or smoothing functions will result in somewhat computationally expensive algorithms.

The aim of this study was to develop and validate a computationally inexpensive algorithm to reliably detect walking in the residential care home environment. We developed both a generic and personalized algorithm to determine if incorporating the individual’s walking data in the processing would improve walking bout identification. To improve robustness of the gait classifier, we made the algorithm independent of the accelerometer orientation, ensuring that improper placement of the accelerometer in the real-world would not lead to significant data loss.

## 2. Materials and Methods

### 2.1. Data Collection

We recruited 27 participants from four long-term care facilities as a sub-study of the “Staying UpRight” study [4]. All participants gave informed consent as approved by the New Zealand (NZ) Health and Disability Ethics Committee (Approval number 18/NTB/151/AM03). Inclusion criteria were people living in long term care and over 65 years of age. Participants were excluded if they received psychogeriatric, respite or palliative care, or were acutely unwell or immobile. We made note of any walking aids the participant used during the study. The demographics are presented in Table 1.

**Figure 1:**
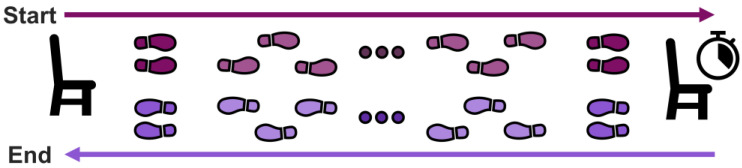
The validation protocol used for this study, wherein participants started seated, stood, walked through a bedroom and hallway, sat down on a chair, then retraced their journey.

At the care home, a trained research assistant attached a 3-axis linear accelerometer (AX3, Axivity Ltd., Newcastle upon Tyne, UK) to the participant using a hydrogel adhesive and covered with a surgical grade adhesive dressing (OPSITE Flexifix™ or Hypafix™, Smith + Nephew Ltd., Watford, UK). The wearable accelerometer was placed in-line with the 5th lumbar vertebrae and orientated such that its long axis was parallel with the spine. Accelerometery data were recorded at 100 Hz with a ±8 g range and accelerometer signals were transformed to a horizontal-vertical coordinate system [48]. The AX3 accelerometer has been validated for its suitability in capturing high-resolution data for long-term analysis [45,49,50]. We first collected a validation dataset with the assistance of two research assistants who were familiar with the care home environment and the participants. The protocol, visualized in Figure 1, took between 10 and 15 min to complete. Data collection began with the participant seated in the care home. The participant was then asked to stand up from their bed, walk along a corridor at their own pace, sit down in a chair, and rest for as long as they wanted, before standing up and retracing their path to the original starting point where they sat down again. Meanwhile, one research assistant used a digital form (Table S1) to record the timings and type of activity. Activities were categorised according to eight specific labels. For the purposes of this study, the two activity categories of “Moving in corridor” and “Moving in confined space” were labelled as walking and all other categories were considered non-walking. The research assistant recorded the clock time (in hour:minute:second format) when the participant began a new type of activity, such as “transition—sit-to-stand”, and also noted the type of activity. Throughout the protocol, a second research assistant used a video camera (HC-V720, Panasonic, Osaka, Japan) with a frame rate of 25 Hz to record the lower half of the participant.

We manually synchronized the accelerometer, research assistant labels, and video data. At the start of the protocol a research assistant aimed the video camera at the wearable sensor on the participant’s back while a mobile phone with the time displayed in hour:minute:second format was held in view of the camera. The second research assistant then tapped the accelerometer three distinct times with their finger to generate a synchronization signal across all datatypes.

We also collected a “real-world” continuous dataset over 7 days using the same wearable sensor set-up, but with no video or hand annotations. Participants wore the sensor throughout the 7-day period and were asked to continue their daily activities as normal. These real-world data were primarily used for the “personalized” algorithm detailed in Section 2.3. Before processing, the 7-day data were cleaned using a custom built MATLAB (v2021b, Mathworks, Nattick, MA, USA) graphic user interface. We could identify and correct for periods wherein the wearable sensor was removed, replaced in a new orientation, or suffered data loss.

### 2.2. Data Annotation

The 3-axis accelerometer data from the validation dataset was aligned with the research assistant labels of “walking” or “non-walking” at every timepoint. We matched the video frames of the wearable sensor being tapped to the corresponding spikes in the sensor’s linear acceleration data. Using the time shown on the mobile phone in the video when the accelerometer was tapped, we aligned the written timings of activity to the accelerometer frames. This 2-step sequence matched the activity labels with the accelerometer frames. We then designated walking activity as binary 1, and all other activity as binary 0.

### 2.3. Algorithm Design

The algorithm identifies periods of walking using thresholds with the magnitude of the linear acceleration in all 3 axes. We developed two versions of the algorithm: one that used a generic threshold and one that used a personalized threshold. There are only two differences between the two algorithms, so we will therefore start with a description of the generic threshold algorithm, hereby referred to as the generic algorithm.

**Figure 2:**
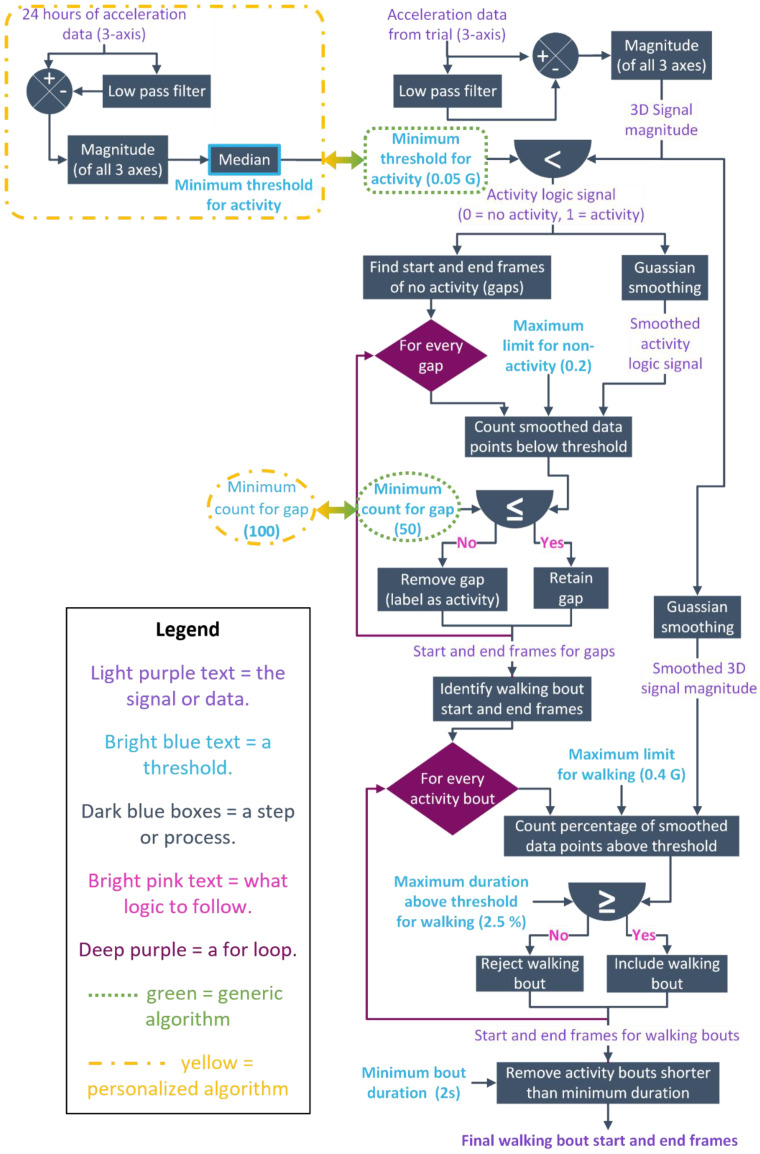
Flow chart showing the steps of the algorithm. The differences between the developed algorithms are shown by the dotted-green shapes, which represents the generic algorithm, and the dot-dash-yellow shape, which illustrates the personalized algorithm.

We described the generic algorithm visually in Figure 2 and provide the code in the supplementary files. We filtered the 3-axes of linear acceleration with a 4th order, zero-lag low pass Butterworth filter with a cut-off frequency of 0.25 Hz, then subtracted the filtered data from the raw data to centre the accelerations around 0 g while maintaining frequency content of the data caused by movement. We calculated the magnitude (or 3D norm) of all three axes to create a single signal indicating overall acceleration, which is compared to the global threshold of 0.05 g. The global threshold is essentially the minimum 3D magnitude at which any type of activity could occur. We determined this threshold through pilot studies with substantial visual inspection on all participants data. Comparison of the 3D signal to the global threshold creates a binary data series where 0 indicates datapoints less than the threshold and 1 represents data exceeding the threshold. The binary signal was smoothed with a Gaussian-weighted moving average filter to provide an estimate of activity likelihood with reference to the previous and future data. The “activity likelihood” signal better captures the possibility of activity by compensating for datapoints which dip below or rise above the global threshold for very short periods of time. A Gaussian filter was used for equal weighting of previous and upcoming data, with closer datapoints having a higher weight than further away datapoints.

We used the binary signal to identify every period of non-walking or “gap”. We then examined the smoothed “activity likelihood” signal during the gap, and counted the number of datapoints less than 0.2 g which was then compared to the “Minimum gap threshold” of 50 frames. If more than 50 datapoints were below the 0.2 threshold, the gap was retained and considered as “no activity”. If less than 20 of the “activity likelihood” datapoints fell below the 0.2 threshold, the gap was removed and the data labelled as “activity”. At this stage, we had the start and end frames of every gap and therefore bout of activity.

The 2nd last stage of the algorithm is to include or reject each identified bout of activity as walking. In each bout, we examined the Gaussian smoothed 3D magnitude of the acceleration data in comparison to a heuristic threshold of 0.4 g. The purpose of this threshold is to remove non-walking activities like sit-stand transitions. If less than 2.5% of the smoothed 3D magnitude data is above the 0.4 g threshold, then the activity is considered walking and the bout is included. Conversely, if more than 2.5% of the data is above the 0.4 g threshold, it is considered non-walking activity and rejected. At the end of this stage, we have the start and end frames for every identified bout of walking.

The final stage of the algorithm rejected walking bouts that were less than the minimum duration. For the purposes of further analysis, we set the minimum walking bout duration to 2 s. Walking bouts shorter than 2 s were rejected. The output of the algorithm was both the start and end frames of each walking bout and a binary signal wherein 1 represents walking and 0 represents non-walking.

In the personalized algorithm we replaced the “generic threshold” with a threshold value determined by the participant’s data and we also modified the “minimum gap threshold”. The minimum threshold for activity was found as the median of the 3D magnitude in a 24 h period. Theoretically, we are more often stationary than we are in movement, therefore the median acceleration value over a 24 h period should indicate the baseline of no movement. In this study, we found the median for each participant from day 3 of the real-world data. We chose to use the data from day 3 to determine the median as no participants suffered data loss or anomalies on this day, and each participant had time to acclimate to the feeling of the sensor on their back. This personalized threshold replaced the “generic” threshold value in the algorithm. The “minimum gap threshold” was set to 100 frames, as pilot testing showed the original value of 50 frames was too conservative when combined with the personalized threshold (see Figure 2.)

### 2.4. Algorithm Validation and Performance Evaluation

To validate and evaluate both versions of the presented algorithm, we included comparison to the research assistant annotations and a previously validated “walking bout detection” algorithm. For the purposes of this manuscript, the research assistant annotated walking bouts were considered as the “ground truth”. The walking bout detection algorithm developed by Hickey et al. [42] was validated with a young, able-bodied population through comparison with chest-mounted camera footage. For simplicity, we will refer to the algorithm developed by Hickey et al. [42] as the “Reference” algorithm.

**Figure 3:**
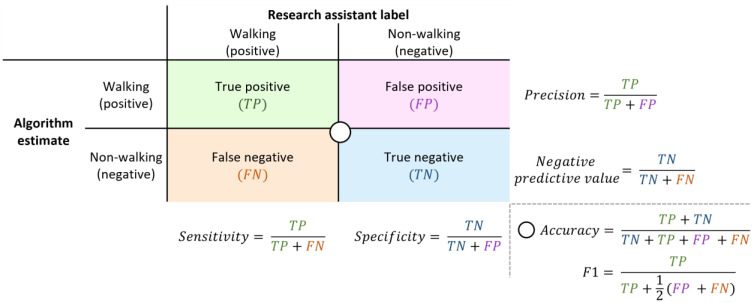
Confusion matrix which defines “true positive”, “true negative”, “false positive”, and “false negative” in the context of research assistant labelled and algorithm estimated data points. The equations used to calculate the sensitivity, specificity, precision, negative predictive value, accuracy, and F1 score are displayed and positioned with reference to their components.

For a comprehensive and quantitative evaluation of the algorithms, we applied traditional evaluation of binary classifiers matrix to compare each of the algorithms to the ground truth. The evaluation metrics were based on the prediction of the algorithm in comparison to the “ground truth” of the research assistant labelled data. Our evaluation of binary classifiers included the basic ratios: sensitivity (true positive rate), specificity (true negative rate), precision (positive predictive value), and negative predictive value. We also calculated the accuracy and F1 score. Figure 3 presents a confusion matrix to illustrate how we calculated the evaluation metrics. For every participant, we ran analysis on the binary output from each of the algorithms and quantified evaluation metrics. We then averaged the results across participants. We chose to average over participants to ensure that the trial duration was not incorporated in the final evaluation and that the effectiveness of the algorithm was weighted equally for each participant.

Finally, we also compared the run time of each of the algorithms. The run time of the codes were measured with MATLAB’s built-in *tic* and *toc* functions. We recorded the run time of each algorithm thrice for each participant’s data and then averaged across the three attempts. We then divided each participants average run time by the number of data points and multiplied by 6000 to determine the processing time per 10 min of data. We used 2-sample t-tests to investigate significant differences in processing time between the algorithms. Finally, we averaged the processing time per minute of data over participants to find the average processing time per 10 min of data (6000 frames) for each of the three algorithms.

## 3. Results

The final dataset used for analysis included 20 participants, the demographics of whom are summarized in Table 1. Seven of the original 27 participants were excluded from data analysis due to issues found in their validation data. Five participants suffered from data loss and were removed from the analysis. A further participant was excluded due to inadequate attachment of the accelerometer, which resulted in the sensor hanging upside down for a portion of the trial. A final participant exhibited highly unusual accelerometer data which resulted in no walking bouts identified by the developed algorithms. The non-standard accelerations were caused by either inadequate attachment of the sensor or the participant’s additional walking aids: an orthotic shoe and ankle orthosis on the right leg. We included their data in the analysis in Tables S2–S4, but excluded the participant in the following analysis due to uncertainty in the cause of the unusual accelerometer data. In some trials, the research assistant mistakenly tapped on the phone, rather than the accelerometer. In these trials, we visually inspected the video and accelerometer data to align the activity labels with the frames of the wearable sensor.

**Figure 4:**
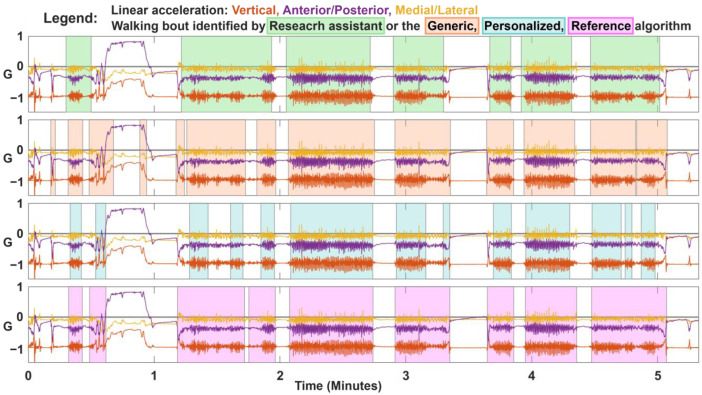
Representative data and walking bout classification from the validation protocol for a single participant. The shaded boxes represent the walking bout as identified by the research assistant (top), and generic, personalized, and reference algorithms (second to fourth plots).

We present representative data of a participant in Figure 4. Visual inspection showed both the generic and personalized algorithms were relatively good at identifying the start and end of each walking bout. However, the generic algorithm sometimes incorrectly considered non-walking activity (i.e., transitions between standing and sitting) to be walking. The personalized algorithm was more conservative than the generic algorithm, which was seen by fewer misclassifications of sit-stand transitions, but increased misclassification of slower walking. The reference algorithm was more likely to classify stationary data as walking compared to the generic and personalized algorithms. Overall, the walking bouts identified by the three algorithms were similar to the research assistant labelled walking bouts.

**Figure 5:**
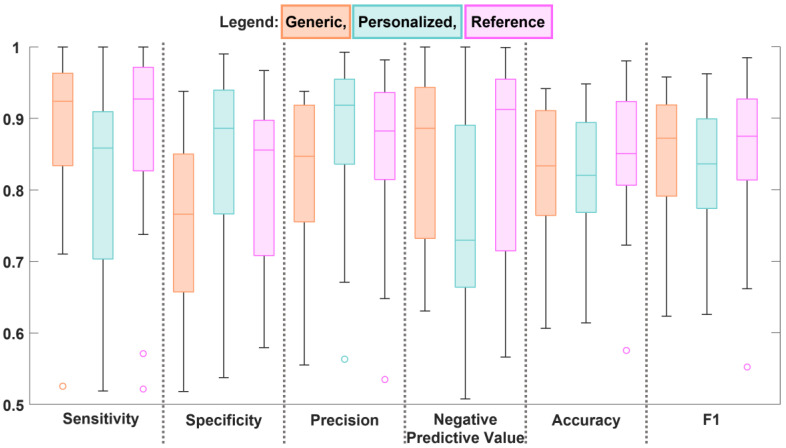
Boxchart of the metrics used to evaluate the efficacy of the walking bout detection algorithms.

We illustrated the quantitative results for each algorithm across participants as a boxchart (Figure 5) and the means with standard deviation for each algorithm in Table 2. The generic algorithm and the references algorithm had very similar sensitivity values (0.90 and 0.89 respectively). The lower sensitivity of the personalized algorithm indicates the algorithm was a little conservative in classifying walking data, in agreement with the finding drawn from Figure 4. The specificity of the generic algorithm was the lowest of the algorithms (0.74), which supports the earlier statement that the generic algorithm was more likely to classify sit-stand transitions as walking. Similarly, the personalized algorithm had the highest specificity of the algorithms (0.84) indicating it had fewer misclassification of sit-stand transitions. Although the personalized algorithm excelled in precision, we found it had the worst negative predictive value. The generic algorithm was the least precise, although it shared mean negative predictive value with the reference algorithm. The accuracy and F1 score were similar across all algorithms, although the box chart indicated the reference algorithm had the least spread for accuracy.

**Figure 6:**
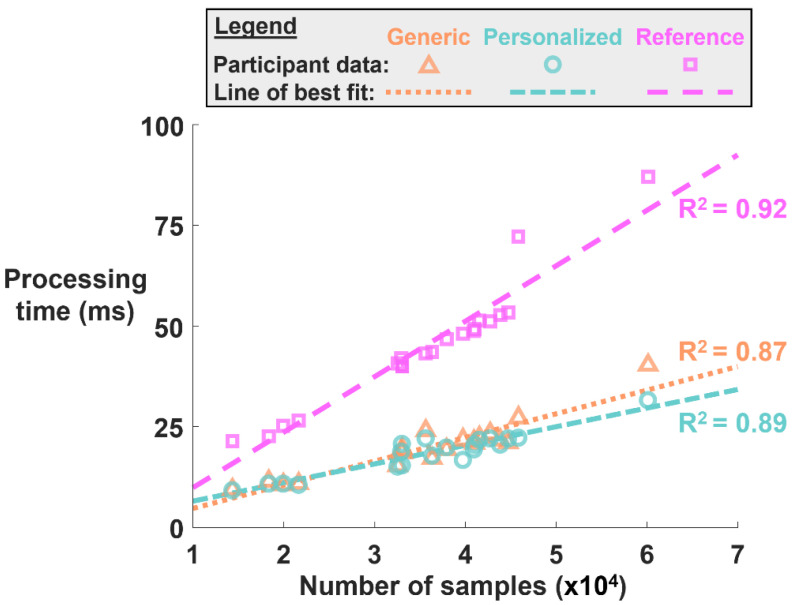
The processing time for each of the algorithms. Each algorithm’s processing time has been fit with the line of best fit (linear regression).

Both the generic and personalized algorithms had significantly shorter processing times than the references algorithm (Figure 6) and Table 3. There was no significant difference in processing time between the generic and personalized algorithms (*p* = 0.50). We found *p* < 0.01 when independently comparing the reference algorithm to generic and personalized algorithm.

## 4. Discussion

The primary goal of this study was to determine if we could accurately and reliably measure walking bouts using computationally efficient algorithm in a heterogenous population of people with mobility limitations living in residential care environments. Our results show that both of our developed algorithms were able to reliably detect walking bouts compared to the ground truth of research assistant labels. Unobtrusively and reliably measuring walking activity of people in long-term care is a valuable tool for understanding disease progression and the impact of interventions, as well as guiding treatment and therapy plans.

We found the developed algorithms performed similarly to the reference algorithm, with some differences between all three. The reference algorithm was the most likely to include non-walking data within correctly identified walking bouts. That is to say, the reference algorithm had the poorest performance at detecting short breaks in walking bouts. Low specificity, or labelling non-walking data as walking, causes noise and issues when determining gait outcomes (such as walking speed, initial contact timings, or step length) because the algorithms will attempt to calculate gait characteristics from data that has none of the characteristics of walking data. The algorithms developed and described in this manuscript were generally very good at excluding non-walking data within walking bouts, and accurately identifying the start and end of confirmed walking bouts. The generic algorithm was the least conservative of the algorithms, which was indicated by high sensitivity and low specificity. The personalized algorithm was the most conservative at classifying walking bouts. The high specificity of the personalized algorithm is highly desirable for extracting gait characteristics as it reduces the noise and errors that are introduced by including non-walking data in the analysis. Otherwise, the results of the three algorithms were somewhat comparable and showed acceptable consensus with research assistant labelling.

All algorithms misclassified some transition data (i.e., sit–stand) as walking. Transitioning between standing and sitting incurs high linear accelerations, which causes misclassifications by all algorithms. Visually, there are no repeating cyclic patterns during transitions as there is in walking. However, none of the presented algorithms look for repeating patterns. The developed algorithms could implement an additional threshold or check to reject transition data as walking. A simple maximum magnitude threshold implemented in tandem with the minimum magnitude threshold may improve rejection of transitions. An alternative technique would be inclusion of gyroscope data, which was not measured by our sensors.

Incorporating gyroscope data with the linear acceleration could improve the performance of both developed algorithms. Specifically, the gyroscope data gives information on trunk orientation, which could be used to represent a facet of walking. Older adults exhibit a trunk flexion angle relatively close to upright when walking (average 6.3 degrees for men, and 7.0 degrees for women) [51]. The trunk orientation could be compared to a personalized threshold to classify if the trunk angle is within normal limits for walking. The position classification would then be included in the processing step to reject non-walking activity in the developed algorithms. The trunk angle threshold may also be derived from participant data, improving the individualization of the classifier. However, including gyroscope data would not only increase the processing time, but require different hardware. We think including gyroscope data would be worthwhile as improved accuracy would be worth a small increase in processing time.

The threshold of the personalized algorithm was found from a separate data set, which may have resulted in unsuitable threshold values. The dataset used for determining the personalized threshold was recorded on a different day, with no guarantee the measurement unit was positioned exactly as it was for the validation data collection. Furthermore, the participant may have had different gait patterns between the data collections. If the threshold was derived from a participant’s “bad” day, then the thresholds may not match with data from a “good” day. An adaptive personalized threshold which is determined from the most recent data may overcome this issue.

All thresholds and limits, except the personalized minimum activity threshold, were determined through human reasoning. In piloting, we tested numerous values for each threshold and limit. Through participant-level visual comparison of the data at every step, we evaluated the various threshold values. We chose the thresholds that appeared to work well with all participants and were neither overly conservative nor generous. It is highly likely that the thresholds and limits were suboptimal. Further investigation into the most appropriate thresholds and limits is warranted. In addition to finding more appropriate generic thresholds, we may also find personalized thresholds more suitable than generic thresholds. In general, the use of adaptive or personalized thresholds increase robustness of the algorithm, which is particularly important for irregular or unstable gait patterns [15,47].

The population for which these algorithms are designed is a heterogenous population, which explains the large spread of classification evaluation metrics. People living in long term care will typically share some gait characteristics such as slow walking. However, the variety of medical conditions and mobility issues that are present in the population mean there is a wide range of gait patterns. The majority of participants included in this study used a walking frame to complete the protocol and yet each of their acceleration patterns were visibly different. We found that the developed algorithms were far more suitable for some participants than others. For example, the developed algorithms were unable to identify walking bouts in the data of participant 21, who walked with a walking frame and orthotic shoe with ankle orthosis on the right leg. We cannot be certain if the developed algorithms performed poorly for this participant due to poor sensor attachment or unusual patterns in the acceleration data. If we were to group the participants by gait characteristics, we may discover which gait metrics improve the efficacy of the algorithms and which gait characteristics are difficult for the algorithm to handle. Unfortunately, we did not measure gait characteristics so cannot perform this analysis.

### 4.1. Limitations

**Figure 7:**
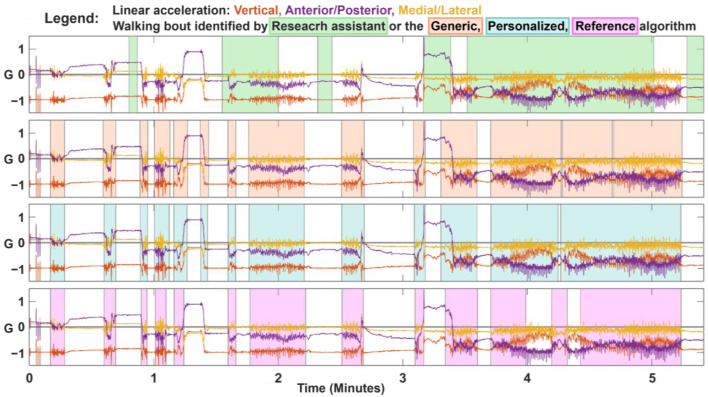
Representative data from the validation protocol of a single participant exhibiting poor match between walking bouts identified by research assistant labels and linear acceleration data.

Due to methodical difficulties, there were apparent inaccuracies in the research-assistant-labelled data that we believe may have impacted the results and subsequent interpretation. We illustrated an example of inaccurate research-assistant-labelled walking bouts in Figure 7. From visual inspection of this participant’s data, we found that the research assistant labelled walking bouts do not overlap with actual periods of walking which we identified by the low frequency fluctuations in linear acceleration. We suggest two likely causes of the inaccuracy: synchronization error, and difficulty cataloguing the precise timings of activities. The former is due to difficulty matching the accelerometer frames to the research assistant labels, which had a few contributing factors. The time shown on the mobile phone in the video data varied in precision, sometimes displaying the time to the nearest second and other times to the closest minute. In trials where the time was shown to the nearest minute, it was not trivial to match the seconds of the research assistant labels to the video and wearable sensor data. In trials where the phone was mistakenly tapped instead of the accelerometer, we visually compared accelerometer data with both the video and research assistant labels. The start and stop times of the walking bouts identified from visual inspection of the linear acceleration may not have matched precisely with the start and stop times of the research assistant labels.

The difficulty of cataloguing the precise timings of the activities in real time manifested in a multiple ways. Firstly, the research assistants noted times to the nearest second, whereas the accelerometer measured time in tenths of a second (0.01 s). Therefore, there is uncertainty as to which activity is being performed for nine frames of the accelerometer data. Furthermore, and perhaps more importantly, it was difficult for the research assistants to notice and record small details in walking bouts due to human reaction times. A participant may take a short break during their walking, which is challenging for the research assistant to note down in real time. In addition, identifying the exact time the participant transitions between activities is very difficult to do visually. For example, the research assistant used their best judgement to determine when the participant started walking from standing. These challenges result in less accurate labelling, which impacts the assessment of the algorithms.

Considering the difficulties in obtaining highly accurate research assistant labelled data, it is possible that we have underestimated the performance of the developed algorithms. The most impactful of these difficulties is the disagreement between the algorithms and labelled data when short breaks in walking occur. Visual inspection of the acceleration and video data shows the algorithms identified true short breaks in some walking bouts that were labelled by the research assistant as walking. As such, a number of true negatives were incorrectly evaluated as false negatives, which primarily implies underestimation of the negative predictive value as well as a slightly underestimated true positive and negative rate. To improve the quality of our evaluation, we could relabel the data using the recorded videos; however, such a task is extremely time intensive and subject to human error and was therefore not included in this study. Future work may find it worthwhile to relabel the data.

### 4.2. Computational Cost and Processing Time

The processing time and therefore computational cost of the developed algorithms were far less than that of the reference algorithm. The main difference in computational cost was likely due to the different techniques used in the initial estimate of walking bouts. The Hickey et al. algorithm initially assessed the data for activity using small windows, requiring multiple repetitions of the analysis technique. Our developed algorithms initially processed the data as a single vector, requiring no repetition or segmentation of the data. Shorter processing time (and lower computational cost) of an algorithm makes it more suitable for on-board processing as it is less draining on the battery and requires fewer resources. Although this is helpful for a personal device to estimate walking at the end of each day, our algorithms would require some modification to estimate walking bouts in real time.

### 4.3. Future Considerations

Adapting our presented algorithms for real-time use is possible, but would require some fundamental changes. It is likely that the data would need to be sectioned for processing. In addition to identifying the optimal window size and style (such as overlapping or adjacent), consideration must be paid to how the Gaussian smoothing and filtering impact the data. New thresholds may be needed to adapt for changes in the processed signal.

Finally, our algorithms are not reliant on the orientation of the wearable sensor but they do necessitate good adhesion to the skin. The algorithm combines all three axes of linear acceleration into one vector, which is used for processing. As such, the algorithm results should be unchanged if the sensor was placed in any other orientation. We have not yet tested this theory. However, it is important that the wearable sensor remains in place—if the accelerometer can move relative to the skin, the linear accelerations will be changed and the algorithms are less likely to properly detect walking bouts.

## 5. Conclusions

In conclusion, our developed algorithms were able to measure walking bouts in a real-world environment for people living in long-term care with acceptable accuracy and reliability. Our generic algorithm was less conservative than the personalized algorithm, but both were able to identify short breaks in walking. Both algorithms were able to identify the start and end of the walking bouts with high accuracy, although sit-to-stand and stand-sit-transitions were sometimes mislabelled as walking. Our results showed good agreement between the developed algorithms, reference algorithm, and ground truth (research-assistant-labelled data), but also highlighted difficulties and inaccuracies of real-time human annotation of activities. We were also able to decrease processing time and computational complexity compared to a previously validated algorithm from Hickey et al. Going forward, we can optimize the generic and personalized thresholds of the algorithms and consider adaptation for real-time implementation.

## Figures and Tables

**Table 1:** Demographics of the participants included in the final data analysis (21 of 27 were included).

Participant	Age (Years)	Height (m)	Weight (kg)	Sex	Walking Aid(s)
1	93	1.58	52	Female	None
2	104	1.68	53	Male	None
3	81	1.57	62	Female	None
4	94	1.65	57	Female	None
5	68	1.65	118	Female	Stick, right hand
6	69	1.75	83	Male	Walking frame
7	77	1.78	109	Male	Walking frame
8	85	1.55	47	Female	Walking frame
9	87	1.61	37	Female	Walking frame
10	88	1.74	82	Male	Walking frame
11	81	1.48	64	Female	Walking frame
12	80	1.78	102	Male	Walking frame
13	83	1.60	56	Female	Walking frame
14	89	1.61	61	Female	Walking frame
15	94	1.71	74	Male	Walking frame
16	97	1.75	68	Male	Walking frame
17	79	1.71	90	Male	Walking frame
18	88	1.73	73	Male	Walking frame
19	81	1.50	52	Female	Walking frame
20	85	1.65	48	Female	Walking frame
21	65	1.54	75	Male	Walking frame, Right Ankle Brace and Orthotic shoe

**Table 2:** The evaluation metrics of the generic, personalized, and reference algorithms, averaged over participants.

	Generic		Personalized		Reference	
	Mean	s.d	Mean	s.d	Mean	s.d
Sensitivity (True Positive Rate)	0.90	0.08	0.81	0.13	0.89	0.11
Specificity (True Negative Rate)	0.74	0.12	0.84	0.13	0.80	0.11
Precision (Positive Predictive Value)	0.82	0.11	0.87	0.11	0.85	0.11
Negative Predictive Value	0.85	0.12	0.77	0.13	0.85	0.14
Accuracy	0.83	0.09	0.82	0.09	0.85	0.09
F1	0.86	0.09	0.83	0.09	0.87	0.10

**Table 3:** Mean processing time per 6000 frames (time is in milliseconds).

	Generic	Personalized	Reference
Mean	3.31	3.13	7.59
s.d.	0.39	0.36	0.64

## Data Availability

The data presented in this study are openly available in GitHub at https://github.com/Rai-Mac/GaitDetection (accessed on 1 November 2023). The data presented in this study are available on request from authors N.K and L.T.

## Supplementary Materials

The following supporting information can be downloaded at: https://www.mdpi.com/article/10.3390/s23218973/s1, Table S1: Data collection form used by research assistants to label and time activities during the experimental protocol; Table S2: Individual results of the developed generic algorithm; Table S3: Individual results of the developed personalized algorithm; Table S4: Individual results of the walking “reference” walking bout detection algorithm from Hickey et al.
