# Supplementary material for: Walking Bout Detection for People Living in Long Residential Care: A Computationally Efficient Algorithm for a 3-Axis Accelerometer on the Lower Back
Source: Sensors (Basel). 2023 Nov 4;23(21):8973. doi: 10.3390/s23218973 (PMC10647554; doi:10.3390/s23218973)
Supplement: Supplementary file 1 [file sensors-23-08973-s001.zip › Table S1.pdf]

Table S1: Data collection form used by research assistants to label and time activities during the experimental protocol.

**Test Date**	
**Accelerometer #**	
**Participant ID**<br>ID Format: NZE_Facility_Participant ID_OBS<br>NZE_XX_XXX_OBS	
**Walking aid used (tick which applies)**	☐Walking frame ☐High walking frame<br>☐Walking stick right hand ☐Walking stick left hand<br>☐None
**Date of Birth**	
**Accelerometer height (metres)**	
**Participant height**	
**Participant weight**	

**Test Start time (Hr:mm:ss) – from the video**	**Start time for each <u>item</u>**	**Activity – (*number the activity in order e.g.,1,2,3) . Not all activities need to occur in each area of the home***	**Notes-** ***tick any item that applies***
**BEDROOM**		☐Transition – stand to sit<br>☐Transition – sit to stand<br>☐Sitting<br>☐Standing<br>☐Moving in bedroom/ confined space<br>☐Transition – stand to lying<br>☐Transition- lying to sit<br>☐Transition – sit to stand	☐ *Had additional help from a person*<br>☐ *Stopped during walking*<br>☐ *Stopped during transfer*<br>☐ *Negotiating an object while walking*<br>☐ *Picking something off a table or similar*<br>☐ *Other (state)________*
**CORRIDOR**		☐Moving in corridor<br>☐Transition – stand to sit<br>☐Sitting<br>☐Transition- sit to stand<br>☐Moving in corridor	☐ *Had additional help from a person*<br>☐ *Stopped during walking 3s*<br>☐ *Stopped during transfer*<br>☐ *Negotiating an object while walking*

			☐ *Picking something off a table or similar*<br>☐ *Other (state)________*
**LOUNGE/DINING**		☐Moving around lounge/dining area<br>☐Transition – stand to sit<br>☐Sitting<br>☐Transition – sit to stand<br>☐Standing still<br>☐Moving around lounge/dining area	☐ *Had additional help from a person*<br>☐ *Stopped during walking*<br>☐ *Stopped during transfer*<br>☐ *Negotiating an object while walking*<br>☐ *Picking something off a table or similar*<br>*Other (state)________*
**CORRIDOR**		☐Moving in corridor<br>☐Transition – stand to sit<br>☐Sitting<br>☐Transition- sit to stand<br>☐Moving in corridor	☐ *Had additional help from a person*<br>☐ *Stopped during walking*<br>☐ *Stopped during transfer*<br>☐ *Negotiating an object while walking*<br>☐ *Picking something off a table or similar*<br>☐ *Other (state)________*
**BEDROOM**		☐Transition – moving in bedroom/ confined space<br>☐Standing still<br>☐Transition – stand to sit<br>☐Sitting<br><br>**FINISH TIME**	☐ *Had additional help from a person*<br>☐ *Stopped during walking*<br>☐ *Stopped during transfer*<br>☐ *Negotiating an object while walking*<br>☐ *Picking something off a table or similar*<br>☐ *Other (state)________*
