# Supplementary material for: Walking Bout Detection for People Living in Long Residential Care: A Computationally Efficient Algorithm for a 3-Axis Accelerometer on the Lower Back
Source: Sensors (Basel). 2023 Nov 4;23(21):8973. doi: 10.3390/s23218973 (PMC10647554; doi:10.3390/s23218973)
Supplement: Supplementary file 1 [file sensors-23-08973-s001.zip › Table S2.pdf]

Table S2: Individual results of the developed generic algorithm.

| Participant | Sensitivity | Specificity | Precision | Negative Predictive Value | Accuracy | F1 |
|---|---|---|---|---|---|---|
| 1 | 0.82 | 0.84 | 0.92 | 0.69 | 0.83 | 0.87 |
| 2 | 0.98 | 0.82 | 0.92 | 0.94 | 0.92 | 0.95 |
| 3 | 0.95 | 0.76 | 0.76 | 0.95 | 0.85 | 0.84 |
| 4 | 0.76 | 0.72 | 0.82 | 0.65 | 0.75 | 0.79 |
| 5 | 0.99 | 0.67 | 0.76 | 0.98 | 0.83 | 0.86 |
| 6 | 0.78 | 0.66 | 0.70 | 0.75 | 0.72 | 0.73 |
| 7 | 0.71 | 0.52 | 0.56 | 0.68 | 0.61 | 0.62 |
| 8 | 0.87 | 0.64 | 0.73 | 0.82 | 0.76 | 0.79 |
| 9 | 0.92 | 0.77 | 0.84 | 0.89 | 0.86 | 0.88 |
| 10 | 0.92 | 0.57 | 0.83 | 0.76 | 0.81 | 0.87 |
| 11 | 0.95 | 0.56 | 0.85 | 0.81 | 0.84 | 0.90 |
| 12 | 0.84 | 0.78 | 0.91 | 0.63 | 0.82 | 0.87 |
| 13 | 0.86 | 0.71 | 0.65 | 0.89 | 0.77 | 0.74 |
| 14 | 0.96 | 0.56 | 0.74 | 0.92 | 0.79 | 0.84 |
| 15 | 1.00 | 0.83 | 0.92 | 1.00 | 0.94 | 0.96 |
| 16 | 0.97 | 0.87 | 0.93 | 0.94 | 0.93 | 0.95 |
| 17 | 0.88 | 0.90 | 0.94 | 0.82 | 0.89 | 0.91 |
| 18 | 0.94 | 0.90 | 0.94 | 0.90 | 0.93 | 0.94 |
| 19 | 0.97 | 0.84 | 0.88 | 0.96 | 0.91 | 0.92 |
| 20 | 0.96 | 0.87 | 0.88 | 0.96 | 0.92 | 0.92 |
| 21 | 0.00 | 0.88 | 0.00 | 0.28 | 0.27 | 0.00 |
| Average | 0.86 | 0.75 | 0.78 | 0.82 | 0.81 | 0.82 |
| Standard deviation | 0.21 | 0.12 | 0.21 | 0.17 | 0.15 | 0.20 |
