# Supplementary material for: Walking Bout Detection for People Living in Long Residential Care: A Computationally Efficient Algorithm for a 3-Axis Accelerometer on the Lower Back
Source: Sensors (Basel). 2023 Nov 4;23(21):8973. doi: 10.3390/s23218973 (PMC10647554; doi:10.3390/s23218973)
Supplement: Supplementary file 1 [file sensors-23-08973-s001.zip › Table S3.pdf]

Table S3: Individual results of the developed personalized algorithm.

Participant	Sensitivity	Specificity	Precision	Negative Predictive Value	Accuracy	F1
1	0.73	0.91	0.94	0.61	0.79	0.83
2	0.96	0.90	0.95	0.92	0.94	0.96
3	0.87	0.83	0.81	0.89	0.85	0.84
4	0.62	0.88	0.89	0.58	0.71	0.73
5	0.94	0.77	0.81	0.92	0.86	0.87
6	0.53	0.90	0.84	0.65	0.71	0.65
7	0.70	0.54	0.56	0.68	0.61	0.63
8	0.68	0.94	0.92	0.73	0.81	0.79
9	0.70	0.93	0.92	0.71	0.80	0.80
10	0.89	0.66	0.85	0.73	0.82	0.87
11	0.89	0.70	0.88	0.71	0.83	0.89
12	0.72	0.82	0.92	0.51	0.74	0.81
13	0.82	0.75	0.67	0.87	0.78	0.74
14	0.91	0.61	0.75	0.84	0.78	0.83
15	1.00	0.85	0.93	1.00	0.95	0.96
16	0.73	0.99	0.99	0.67	0.82	0.84
17	0.86	0.95	0.96	0.80	0.89	0.91
18	0.93	0.95	0.97	0.90	0.94	0.95
19	0.86	0.96	0.96	0.85	0.91	0.91
20	0.91	0.89	0.88	0.91	0.90	0.90
21	0.00	0.89	0.00	0.28	0.27	0.00
Average	0.77	0.84	0.83	0.75	0.80	0.79
Standard deviation	0.22	0.12	0.22	0.17	0.15	0.20
