# Supplementary material for: Walking Bout Detection for People Living in Long Residential Care: A Computationally Efficient Algorithm for a 3-Axis Accelerometer on the Lower Back
Source: Sensors (Basel). 2023 Nov 4;23(21):8973. doi: 10.3390/s23218973 (PMC10647554; doi:10.3390/s23218973)
Supplement: Supplementary file 1 [file sensors-23-08973-s001.zip › Table S4.pdf]

Table S4: Individual results of the walking "reference" walking bout detection algorithm from Hickey et al.

Participant	Sensitivity	Specificity	Precision	Negative Predictive Value	Accuracy	F1
1	0.85	0.87	0.93	0.72	0.85	0.89
2	0.97	0.91	0.96	0.93	0.95	0.96
3	0.93	0.72	0.72	0.93	0.81	0.82
4	0.74	0.86	0.89	0.66	0.78	0.81
5	0.98	0.71	0.78	0.97	0.85	0.87
6	0.76	0.86	0.85	0.78	0.81	0.80
7	0.57	0.58	0.53	0.61	0.58	0.55
8	0.88	0.80	0.82	0.86	0.84	0.85
9	0.94	0.84	0.88	0.91	0.89	0.91
10	0.93	0.69	0.87	0.81	0.85	0.90
11	0.88	0.65	0.87	0.69	0.82	0.88
12	0.77	0.84	0.93	0.57	0.79	0.84
13	0.92	0.69	0.65	0.93	0.78	0.76
14	0.97	0.62	0.77	0.94	0.82	0.86
15	1.00	0.88	0.94	1.00	0.96	0.97
16	0.99	0.97	0.98	0.98	0.98	0.98
17	0.87	0.96	0.97	0.81	0.90	0.92
18	0.97	0.90	0.94	0.95	0.94	0.96
19	0.98	0.90	0.92	0.98	0.94	0.95
20	0.97	0.87	0.88	0.97	0.92	0.92
21	0.99	0.90	0.96	0.97	0.96	0.97
Average	0.90	0.81	0.86	0.86	0.86	0.87
Standard deviation	0.11	0.11	0.11	0.13	0.09	0.10
